# The association of neutrophil-to-lymphocyte ratio with post-chemotherapy pulmonary infection in lung cancer patients

**DOI:** 10.3389/fmed.2025.1559702

**Published:** 2025-04-09

**Authors:** Tao Sun, Xiaobo He, Jun Liu

**Affiliations:** ^1^Department of Hematology and Oncology Laboratory, The Central Hospital of Shaoyang, Shaoyang, China; ^2^Department of Scientific Research, The First Affiliated Hospital of Shaoyang University, Shaoyang, China

**Keywords:** neutrophil-to-lymphocyte ratio, lung cancer, post-chemotherapy pulmonary infection, SMOTE, RCS analysis, subgroup analysis

## Abstract

**Background:**

Lung cancer patients are particularly vulnerable to pulmonary infections following chemotherapy, which can lead to suboptimal treatment outcomes and increased mortality rates. The neutrophil-to-lymphocyte ratio (NLR), an established inflammatory marker, has been extensively studied; however, its diagnostic value in identifying post-chemotherapy pulmonary infection (PCPI) in lung cancer patients remains unclear. This study aims to evaluate the independent diagnostic effectiveness of NLR in detecting PCPI among lung cancer patients.

**Methods:**

A retrospective analysis was performed on clinical data from 638 lung cancer patients who underwent chemotherapy at the Central Hospital of Shaoyang between January 2020 and December 2023. After excluding cases with incomplete data, 502 patients were included in the final analysis. Due to the low incidence of PCPI within this cohort (19.52%), the Synthetic Minority Over-sampling Technique (SMOTE) was utilized to achieve data balance. Both the balanced and unbalanced datasets were subsequently analyzed and validated using multivariable regression analysis, restricted cubic spline (RCS) analysis, subgroup analysis, and sensitivity analysis.

**Results:**

The findings demonstrated that NLR serves as an independent risk factor for PCPI in patients with lung cancer, irrespective of dataset balance [balanced dataset: odds ratio (OR) = 1.12, 95% confidence interval (CI): 1.08–1.16; unbalanced dataset: OR = 1.08, 95% CI: 1.03–1.13]. Furthermore, in the balanced dataset, after adjusting for all covariates (Model 4), quartile analysis of NLR revealed a significant increase in the risk of PCPI with higher NLR levels (fourth quartile group OR = 5.64, 95% CI: 3.17–10.01, *p* < 0.001). The RCS analysis corroborated the nonlinear association between NLR and PCPI. Subgroup analysis revealed that within the chemotherapy regimen subgroups, the association between NLR and PCPI was significantly higher in patients receiving platinum-based chemotherapy (PBC) compared to those receiving non-platinum-based chemotherapy (NPBC) (*p* for interaction = 0.001). Sensitivity analyses further affirmed the robustness of the model outcomes.

**Conclusion:**

The analysis in this study indicates that NLR has the potential to be a predictor of PCPI for lung cancer patients. Although these preliminary research findings demonstrate diagnostic promise, its clinical applicability still needs to be verified through multicenter prospective studies to provide reliable evidence for decision-making.

## 1 Introduction

Lung cancer, acknowledged as one of the most prevalent and fatal malignancies globally, has witnessed a continuous rise in both incidence and mortality rates in recent years (1, 2). Despite notable advancements in contemporary medicine, which have introduced a range of therapeutic strategies such as chemotherapy, targeted therapy, and immunotherapy that significantly extend the survival of lung cancer patients, secondary pulmonary infections induced by chemotherapy remain a critical factor adversely affecting patients’ quality of life and prognosis (3, 4). Chemotherapy, although effective in inhibiting tumor progression, can significantly compromise the immune system, particularly impacting the function and quantity of neutrophils and lymphocytes (5–7). These cells are essential components of the body’s immune defense mechanisms, and alterations in their ratio often indicate disruptions in immune status (8). Consequently, lung cancer patients are vulnerable to immunosuppression during chemotherapy, which increases the risk of post-chemotherapy pulmonary infection (PCPI) (9–11). Therefore, early diagnosis and intervention of such infections are imperative for enhancing patient prognosis. Effectively assessing the risk of PCPI in lung cancer patients has become an important issue that urgently needs to be addressed in clinical treatment.

Neutrophil-to-lymphocyte ratio (NLR) has emerged as a significant biomarker reflecting systemic inflammatory response and immune status, attracting considerable attention from the research community in recent years (12–14). NLR is derived from the ratio of neutrophils to lymphocytes, which are measured through routine blood tests. Its simplicity, cost-effectiveness, and accessibility have facilitated its widespread use in clinical research across a range of diseases, including malignancies, cardiovascular disorders, and diabetes (15–17). Despite the promising prognostic potential of NLR in various pathologies, its specific role in post-chemotherapy pulmonary infections (PCPI) in lung cancer patients remains underexplored. Current studies suggest that changes in neutrophil and lymphocyte levels following chemotherapy in lung cancer patients may be closely associated with immune system functionality, potentially affecting the onset and progression of pulmonary infections (18–20). Therefore, investigating the diagnostic utility of NLR in PCPI among lung cancer patients holds substantial clinical importance.

This study seeks to assess the independent diagnostic value of NLR in PCPI in patients with lung cancer and to explore its potential for clinical application. Utilizing a retrospective analysis of clinical data from lung cancer patients who underwent chemotherapy at the Central Hospital of Shaoyang between January 2020 and December 2023, the study initially addressed data imbalance—stemming from the relatively low incidence of pulmonary infections (19.52%)—through data cleaning and Synthetic Minority Over-sampling Technique (SMOTE) oversampling methods. Subsequently, a comprehensive evaluation of factors associated with post-chemotherapy infections in lung cancer patients was performed using multivariable regression analysis, restricted cubic spline (RCS) analysis, subgroup analysis, and sensitivity analysis, particularly emphasizing whether NLR can function as an independent risk predictor. Moreover, the integration of subgroup and sensitivity analyses facilitates further validation of the robustness and reliability of NLR across diverse clinical contexts. Through these systematic analyses, this study aims to clarify the diagnostic role of NLR in PCPI for lung cancer patients and provide a scientific basis for its further promotion in clinical applications.

## 2 Materials and methods

### 2.1 Data source

**Figure 1:**
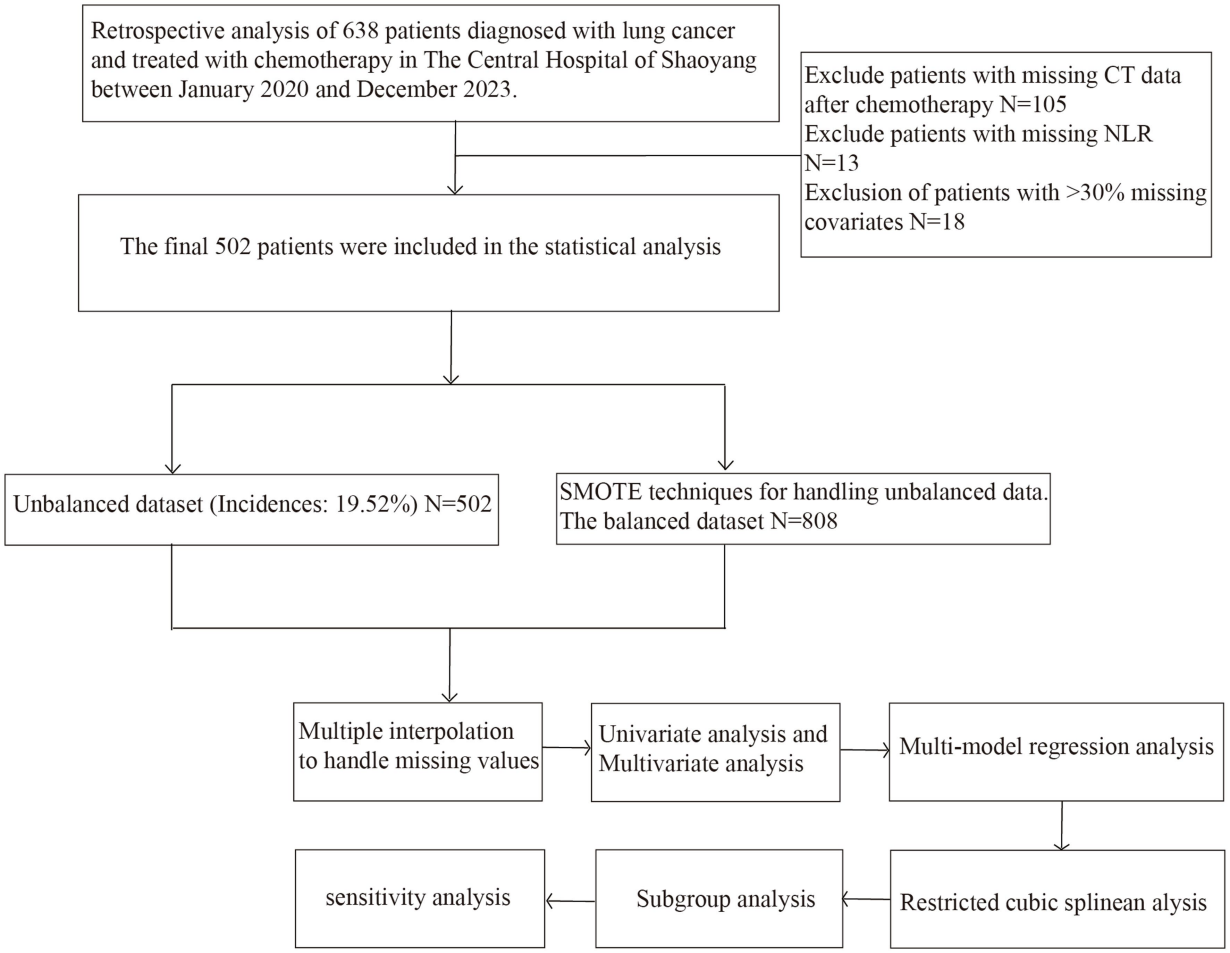
Research flowchart.

This investigation employs a retrospective cohort study design, utilizing data from lung cancer patients who underwent chemotherapy at the Central Hospital of Shaoyang between January 2020 and December 2023. Initially, 638 lung cancer patients satisfied the study’s eligibility criteria. The inclusion criteria were defined as follows: (1) patients with a pathological diagnosis of lung cancer; (2) patients who had completed at least one cycle of chemotherapy; (3) patients possessing comprehensive clinical data, including blood routine tests, imaging studies, and clinical follow-up records; and (4) patients capable of providing blood routine test results both prior to and following treatment. The exclusion criteria encompassed (1) patients experiencing severe immunosuppression prior to chemotherapy; (2) patients with incomplete basic information or clinical data necessary for the study; (3) patients with lung infections present prior to chemotherapy; and (4) patients with tumors located in other regions of the body. Following the exclusion of cases with missing data and those not meeting the inclusion and exclusion criteria, Since the diagnosis of PCPI is primarily based on computed tomography (CT) imaging, patients who did not undergo post-treatment CT scanning were excluded to ensure accurate classification, 502 patients were included in the final analysis. Among these, 98 patients developed lung infections after chemotherapy, representing 19.52% of the cohort. In statistical analysis, considering the low incidence of lung infection events, the SMOTE oversampling technique was used to balance the unbalanced data and reduce the impact of category imbalance on the analysis results (21). Figure 1 shows the detailed data processing and technology roadmap.

### 2.2 Study variables

The primary outcome of this study was the incidence of PCPI. Participants were categorized into two groups based on the diagnosis of lung infection post-chemotherapy: the PCPI group and the non-post chemotherapy pulmonary infection (NPCPI) group. We diagnose PCPI based on clinical symptoms and imaging examination results. The clinical symptoms include fever (≥38°C), cough, increased sputum secretion, dyspnea, or pleural inflammatory chest pain. All patients underwent chest CT, and diagnosis was confirmed by the presence of new or progressive lung infiltrates, comorbidities, or ground-glass opacity. Two independent radiologists reviewed the CT scans to ensure consistency. The primary independent variable investigated was NLR, derived from routine blood tests and calculated as the ratio of the neutrophil count to the lymphocyte count. Additional covariates included fundamental clinical characteristics of the patients, such as gender, age, smoking history, cancer stage, chemotherapy regimen, C-reactive protein (CRP) level, and medical history. We categorized chemotherapy regimens into PBC and NPBC according to the type of chemotherapeutic agent. These variables may influence the risk of infection in patients and, therefore, were controlled for in the multivariate analysis. Supplementary Table 1 shows specific variable information, and Supplementary Figure 1 displays the missing rate of each variable. We found that the missing rates of PCT and CRP reached 84.1 and 74.1%, respectively. Thus, we excluded these two variables from subsequent analyses.

### 2.3 Statistical methods

All statistical analyses were conducted using R software (version 4.2.2) in our study. For comparisons between groups involving normally distributed continuous variables, Welch’s *t*-test or analysis of variance (ANOVA) was employed. In contrast, the Wilcoxon rank-sum or Kruskal–Wallis test was utilized for non-normally distributed variables. For categorical data, Fisher’s exact test was applied when the expected frequency was less than 5; otherwise, the chi-square test was used. Univariate logistic regression analysis was performed to assess the association between NLR and other clinical characteristics (e.g., age, gender, and cancer stage) with the occurrence of PCPI. Factors potentially associated with PCPI were initially screened by calculating odds ratios (ORs) and their 95% confidence intervals (CIs). Based on the univariate analysis, a backward stepwise regression approach, specifically utilizing the stepAIC function from the MASS package in R, was employed to select the optimal combination of variables for inclusion in the multivariate analysis model. Multivariate logistic regression was performed to assess the association between NLR and PCPI, adjusting for gender, alcohol consumption, smoking, hypertension, CHD, radiotherapy, pleural effusion, and other relevant covariates. The potential nonlinear association between NLR changes and clinical outcomes was examined utilizing a logistic regression model incorporating RCS. Various knot configurations, ranging from 3 to 7, were evaluated, and the model exhibiting the lowest Akaike information criterion (AIC) value was selected as the optimal RCS. A subgroup analysis was conducted to evaluate the consistency and stability of NLR across diverse clinical subgroups. Patients were categorized into multiple subgroups based on variables such as gender, age, cancer stage, and history of chronic diseases, and the association between NLR and PCPI was analyzed within each subgroup. To further ensure the robustness and reliability of the findings, a sensitivity analysis was performed by excluding patients with missing data to determine any significant alterations in the primary outcomes. All statistical tests were two-tailed, with a *p*-value of less than 0.05 considered indicative of statistical significance.

## 3 Results

### 3.1 Baseline characteristics of patients

In this study’s initial analysis of baseline data, we assessed various baseline characteristics across balanced and unbalanced datasets and performed statistical evaluations. Table 1 illustrates the distribution of characteristics within the balanced dataset (*N* = 808). No statistically significant differences were identified between the NPCPI and PCPI groups concerning age, BMI, history of diabetes, surgical history, tumor stage, and tumor type (*p* > 0.05). Conversely, significant differences were noted between the groups in variables such as chemotherapy cycles, number of hospitalizations, gender, alcohol consumption, smoking, heart disease, and hypertension (*p* < 0.01). For example, the median (IQR) number of chemotherapy cycles in the PCPI group was 6.00 (4.00, 10.00), significantly exceeding that of the NPCPI group, which was 3.00 (1.00, 5.00). In terms of hospitalizations, the PCPI group exhibited a median (IQR) of 9.00 (6.00, 15.00), markedly higher than the 4.00 (2.00, 7.00) observed in the NPCPI group. Regarding gender distribution, the PCPI group had a higher proportion of males (91.34%), while the NPCPI group had a higher proportion of females (22.03%). In addition, the prevalence of alcohol consumption (16.83%) and smoking (49.75%) was significantly greater in the PCPI group compared to the NPCPI group (6.44 and 20.05%, respectively). The incidence of other conditions, such as hypertension and heart disease, was also markedly higher in the PCPI group relative to the NPCPI group. Supplementary Table 2 presents the baseline characteristics of the unbalanced dataset (*N* = 502), revealing that the analysis results were generally consistent with those of the balanced dataset. Notable differences were identified in the distributions of alcohol consumption, smoking, heart disease, and radiotherapy. In the unbalanced dataset, the PCPI group exhibited higher proportions of smoking (48.98%) and alcohol consumption (19.39%), as well as an increased proportion of radiotherapy (26.53%), compared to the balanced dataset. Additionally, the number of hospitalizations was more significant in the PCPI group than in the NPCPI group, with median values of 10.00 (interquartile range: 5.25, 15.00) versus 4.00 (interquartile range: 2.00, 7.00), respectively. In Supplementary Tables 3, 4, we analysed stratified baseline characteristics according to NLR quartiles. Within the balanced dataset, significant variations were observed in BMI, chemotherapy cycles, number of hospitalizations, and sex as NLR quartiles increased, with *p*-values of <0.001, 0.007, 0.044, and 0.029, respectively. Notably, there were pronounced differences in BMI and the number of hospitalizations between the high NLR group (Q4) and the low NLR group (Q1). Specifically, BMI values were significantly lower in the higher NLR group (Q4) compared to the lower NLR group (Q1). Furthermore, the proportion of males was significantly more significant in the higher NLR group (90.59%) than in the lower NLR group (83.92%). Similar patterns were identified in the unbalanced dataset, where significant differences in BMI, number of hospitalizations, and radiotherapy were observed across different NLR groups (*p*-values <0.001). In particular, BMI was significantly lower in the lower NLR group (Q3) compared to the other groups.

**Table 1:** Baseline information sheet for balanced dataset.

Variables	Outcome			*p*-value
	Overall, *N* = 808^1^	NPCPI, *N* = 404^1^	PCPI, *N* = 404^1^	
Age	65.00 (58.00, 70.00)	65.00 (58.00, 71.00)	65.00 (59.00, 69.00)	0.987^2^
BMI	21.64 (19.87, 23.60)	21.80 (19.53, 24.13)	21.55 (20.09, 23.32)	0.497^2^
Chemotherapy cycle	4.00 (2.00, 8.00)	3.00 (1.00, 5.00)	6.00 (4.00, 10.00)	<0.001^2^
Number of hospitalizations	6.00 (3.00, 11.00)	4.00 (2.00, 7.00)	9.00 (6.00, 15.00)	<0.001^2^
Sex				<0.001^3^
Female	124 (15.35%)	89 (22.03%)	35 (8.66%)	
Male	684 (84.65%)	315 (77.97%)	369 (91.34%)	
Drink				<0.001^3^
No	714 (88.37%)	378 (93.56%)	336 (83.17%)	
Yes	94 (11.63%)	26 (6.44%)	68 (16.83%)	
Smoke				<0.001^3^
No	526 (65.10%)	323 (79.95%)	203 (50.25%)	
Yes	282 (34.90%)	81 (20.05%)	201 (49.75%)	
Diabetes				0.311^3^
No	740 (91.58%)	366 (90.59%)	374 (92.57%)	
Yes	68 (8.42%)	38 (9.41%)	30 (7.43%)	
Hypertension				0.011^3^
No	613 (75.87%)	322 (79.70%)	291 (72.03%)	
Yes	195 (24.13%)	82 (20.30%)	113 (27.97%)	
CHD				<0.001^3^
No	762 (94.31%)	370 (91.58%)	392 (97.03%)	
Yes	46 (5.69%)	34 (8.42%)	12 (2.97%)	
Surgery				0.196^3^
No	710 (87.87%)	349 (86.39%)	361 (89.36%)	
Yes	98 (12.13%)	55 (13.61%)	43 (10.64%)	
Radiotherapy				<0.001^3^
No	631 (78.09%)	341 (84.41%)	290 (71.78%)	
Yes	177 (21.91%)	63 (15.59%)	114 (28.22%)	
Stage				0.131^3^
I stage	25 (3.09%)	18 (4.46%)	7 (1.73%)	
II stage	82 (10.15%)	43 (10.64%)	39 (9.65%)	
III stage	334 (41.34%)	160 (39.60%)	174 (43.07%)	
IV stage	367 (45.42%)	183 (45.30%)	184 (45.54%)	
Typing				0.101^3^
Adenocarcinoma	370 (45.79%)	189 (46.78%)	181 (44.80%)	
Squamous	282 (34.90%)	134 (33.17%)	148 (36.63%)	
SCLC	144 (17.82%)	71 (17.57%)	73 (18.07%)	
Others	12 (1.49%)	10 (2.48%)	2 (0.50%)	
Pleural effusion				<0.001^3^
No	609 (75.37%)	353 (87.38%)	256 (63.37%)	
Yes	199 (24.63%)	51 (12.62%)	148 (36.63%)	
Chemotherapy regimen				0.939^3^
PBC	563 (69.68%)	281 (69.55%)	282 (69.80%)	
NPBC	245 (30.32%)	123 (30.45%)	122 (30.20%)	
NLR	4.35 (2.88, 7.04)	3.73 (2.52, 6.00)	4.81 (3.43, 8.14)	<0.001^2^

1 Median (IQR); *n* (%).

2 Wilcoxon rank sum test.

3 Pearson’s chi-squared test.

### 3.2 Independent clinical factors influencing post-chemotherapy lung infection

**Figure 2:**
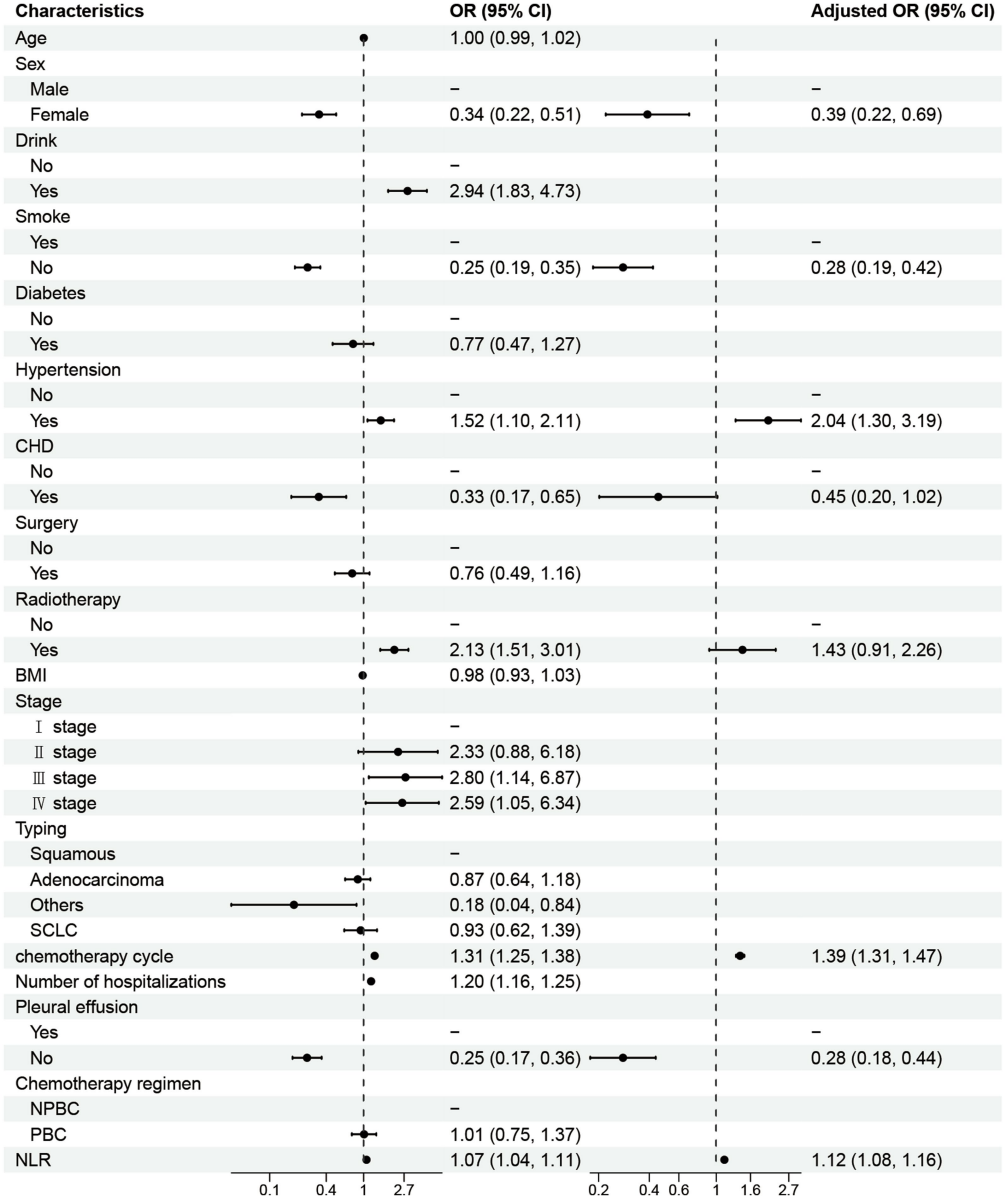
Univariate and multivariate logistic regression analysis forest plots.

In this study, NLR was identified as a significant predictor of post-chemotherapy lung infection risk in both balanced and unbalanced datasets. Utilizing univariate and multivariate logistic regression analyses, we identified both consistencies and discrepancies in NLR’s predictive value across the two datasets. In the balanced dataset (Figure 2), the univariate analysis revealed a significant positive correlation between NLR and lung infection risk (OR = 1.07, 95% CI: 1.04–1.11, *p* < 0.001), indicating that each unit increase in NLR corresponds to a 7% increase in the risk of post-chemotherapy lung infection. This finding was corroborated by multivariate analysis, which established NLR as an independent risk factor for lung infection (OR = 1.12, 95% CI: 1.08–1.16, *p* < 0.001), even after adjusting for confounding variables such as chemotherapy cycles and hospitalizations. These results highlight NLR’s efficacy as an inflammatory marker that reflects the body’s immune response status, thereby influencing the likelihood of lung infections. As depicted in Supplementary Figure 2, NLR exhibited a statistically significant positive correlation within the unbalanced dataset (OR = 1.08, 95% CI: 1.03–1.13). While this finding is consistent with the results obtained from the balanced dataset, the effect size is somewhat diminished. This variation may be ascribed to the differing distribution characteristics of the datasets, particularly in contexts where sample size and event frequency are uneven, thereby influencing the effect of NLR. Nonetheless, NLR remained statistically significant in multivariable analysis, suggesting potential predictive value for lung infection risk in this single-center cohort, though its generalizability requires validation in balanced, prospective studies.

### 3.3 Investigation of the relationship between NLR and post-chemotherapy lung infection under different models

We investigated the relationship between NLR and the incidence of post-chemotherapy lung infection, assessing the influence of NLR across various analytical models. As illustrated in Table 2, a significant association between NLR and post-chemotherapy lung infection was identified in both balanced and unbalanced datasets, corroborated by different regression analyses. In the balanced dataset, NLR, treated as a continuous variable, exhibited a significant positive association with the likelihood of post-chemotherapy lung infection (OR = 1.07, 95% CI: 1.04–1.11, *p* < 0.001). This association was consistently observed across multiple models. In Models 2, 3, and 4, the effect of NLR remained significant, with progressively increasing OR values of 1.08 (95% CI: 1.05–1.11, *p* < 0.001), 1.08 (95% CI: 1.05–1.11, *p* < 0.001), and 1.11 (95% CI: 1.07–1.16, *p* < 0.001), respectively. The findings indicate a strong correlation between an increased NLR and a heightened risk of lung infection. Furthermore, when NLR was standardized, the model analysis results substantiated this association. The OR values for standardized NLR across Models 1 to 4 were 1.67 (95% CI: 1.36–2.05, *p* < 0.001), 1.71 (95% CI: 1.37–2.12, *p* < 0.001), 1.72 (95% CI: 1.37–2.16, *p* < 0.001), and 2.17 (95% CI: 1.65–2.85, *p* < 0.001), respectively, suggesting a more substantial effect of standardized NLR on the incidence of post-chemotherapy lung infection. The quartile analysis indicated a significant upward trend in the risk of pulmonary infection with increasing NLR levels. In the balanced dataset, after adjusting for all covariates (Model 4), the risk of pulmonary infection significantly increased as NLR rose from the first quartile (Q2) to the fourth quartile (Q4) (Q2: OR = 2.13, 95% CI: 1.24–3.66, *p* = 0.006; Q3: OR = 2.70, 95% CI: 1.54–4.74, *p* < 0.001; Q4: OR = 5.64, 95% CI: 3.17–10.01, *p* < 0.001). This trend demonstrated strong consistency across all models (*p* for trend <0.001).

**Table 2:** Association between NLR and PCPI in different modes.

Variables	Model 1		Model 2		Model 3		Model 4	
	OR (95% CI)^1^	*p*	OR (95% CI)^1^	*p*	OR (95% CI)^1^	*p*	OR (95% CI)^1^	*p*
Unbalanced data								
NLR (continuous)	1.07 (1.03–1.11)	<0.001	1.07 (1.03–1.11)	<0.001	1.07 (1.02–1.11)	0.001	1.07 (1.02–1.13)	0.004
NLR (standardized)	1.51 (1.21–1.88)	<0.001	1.49 (1.18–1.88)	<0.001	1.49 (1.16–1.90)	0.001	1.55 (1.15–2.11)	0.004
NLR								
Q1	—		—		—		—	
Q2	0.83 (0.41–1.71)	0.620	0.97 (0.46–2.06)	0.946	0.92 (0.43–1.95)	0.822	0.82 (0.34–1.95)	0.652
Q3	1.54 (0.80–2.93)	0.194	1.52 (0.76–3.05)	0.233	1.50 (0.75–3.01)	0.254	1.32 (0.57–3.03)	0.517
Q4	2.25 (1.21–4.20)	0.011	2.42 (1.25–4.68)	0.009	2.28 (1.17–4.46)	0.016	2.58 (1.13–5.89)	0.025
*p* for trend		0.002		0.003		0.005		0.011
Balanced data								
NLR (continuous)	1.07 (1.04–1.11)	<0.001	1.08 (1.05–1.11)	<0.001	1.08 (1.05–1.11)	<0.001	1.11 (1.07–1.16)	<0.001
NLR (standardized)	1.67 (1.36–2.05)	<0.001	1.71 (1.37–2.12)	<0.001	1.72 (1.37–2.16)	<0.001	2.17 (1.65–2.85)	<0.001
NLR								
Q1	—		—		—		—	
Q2	2.38 (1.58–3.57)	<0.001	2.66 (1.72–4.12)	<0.001	2.59 (1.67–4.02)	<0.001	2.13 (1.24–3.66)	0.006
Q3	2.76 (1.83–4.16)	<0.001	2.66 (1.71–4.14)	<0.001	2.60 (1.67–4.05)	<0.001	2.70 (1.54–4.74)	<0.001
Q4	3.83 (2.53–5.80)	<0.001	4.07 (2.61–6.34)	<0.001	3.79 (2.41–5.95)	<0.001	5.64 (3.17–10.01)	<0.001
*p* for trend		<0.001		<0.001		<0.001		<0.001

1 OR, odds ratio; CI, confidence interval. Model 1: No covariates were adjusted. Model 2: Adjusted for sex, age, drink, smoke, and BMI. Model 3: Adjusted for sex, age, drink, smoke, diabetes, hypertension, CHD, and BMI. Model 4: Adjusted for sex, age, drink, smoke, diabetes, hypertension, CHD, surgery, radiotherapy, BMI, stage, typing, chemotherapy cycle, number of hospitalizations, pleural effusion, and chemotherapy regimen.

In the unbalanced dataset, NLR demonstrated a significant positive correlation with post-chemotherapy lung infection, albeit with a comparatively weaker effect. The OR values for NLR as a continuous variable were consistently 1.07 (95% CI: 1.03–1.11, *p* < 0.001), 1.07 (95% CI: 1.03–1.11, *p* < 0.001), 1.07 (95% CI: 1.02–1.11, *p* = 0.001), and 1.07 (95% CI: 1.02–1.13, *p* = 0.004) across all models, indicating a positive association. Although the effect was slightly attenuated compared to the balanced dataset, it remained statistically significant. Upon standardization, the influence of NLR across different models was reflected in OR values of 1.51 (95% CI: 1.21–1.88, *p* < 0.001), 1.49 (95% CI: 1.18–1.88, *p* < 0.001), 1.49 (95% CI: 1.16–1.90, *p* = 0.001), and 1.55 (95% CI: 1.15–2.11, *p* = 0.004), which were consistent with the findings from the balanced dataset and remained statistically significant. Quartile analysis revealed a more dispersed effect of NLR quartiles in the unbalanced dataset, particularly with lower OR values for the second and third quartiles (Q2 and Q3) that did not achieve statistical significance. However, the OR value for Q4 was 2.25 (95% CI: 1.21–4.20, *p* = 0.011), showing a significant increasing trend across different models, further validating the role of NLR in the unbalanced dataset, albeit with a slightly diminished effect (*p* for trend 0.002 to 0.011). The results suggest that NLR is closely associated with post-chemotherapy lung infection in both balanced and unbalanced datasets.

### 3.4 The nonlinear relationship between NLR and PCPI

To further explore the relationship between NLR and PCPI, we utilized the RCS analysis to assess its potential nonlinear association. Initially, we experimented with varying numbers of knots (3, 4, 5, 6, and 7) to fit the RCS model and computed each configuration’s AIC value. The findings revealed that the model incorporating four knots produced the lowest AIC value (AIC = 747.91). Consequently, we selected the 4-knot model for subsequent analysis. In this model, the knots were strategically positioned at the 5th, 35th, 65th, and 95th percentiles of the independent variable values across all patients. These knots delineated distinct ranges of NLR values, thereby facilitating the evaluation of NLR’s impact on the risk of PCPI across various intervals.

**Figure 3:**
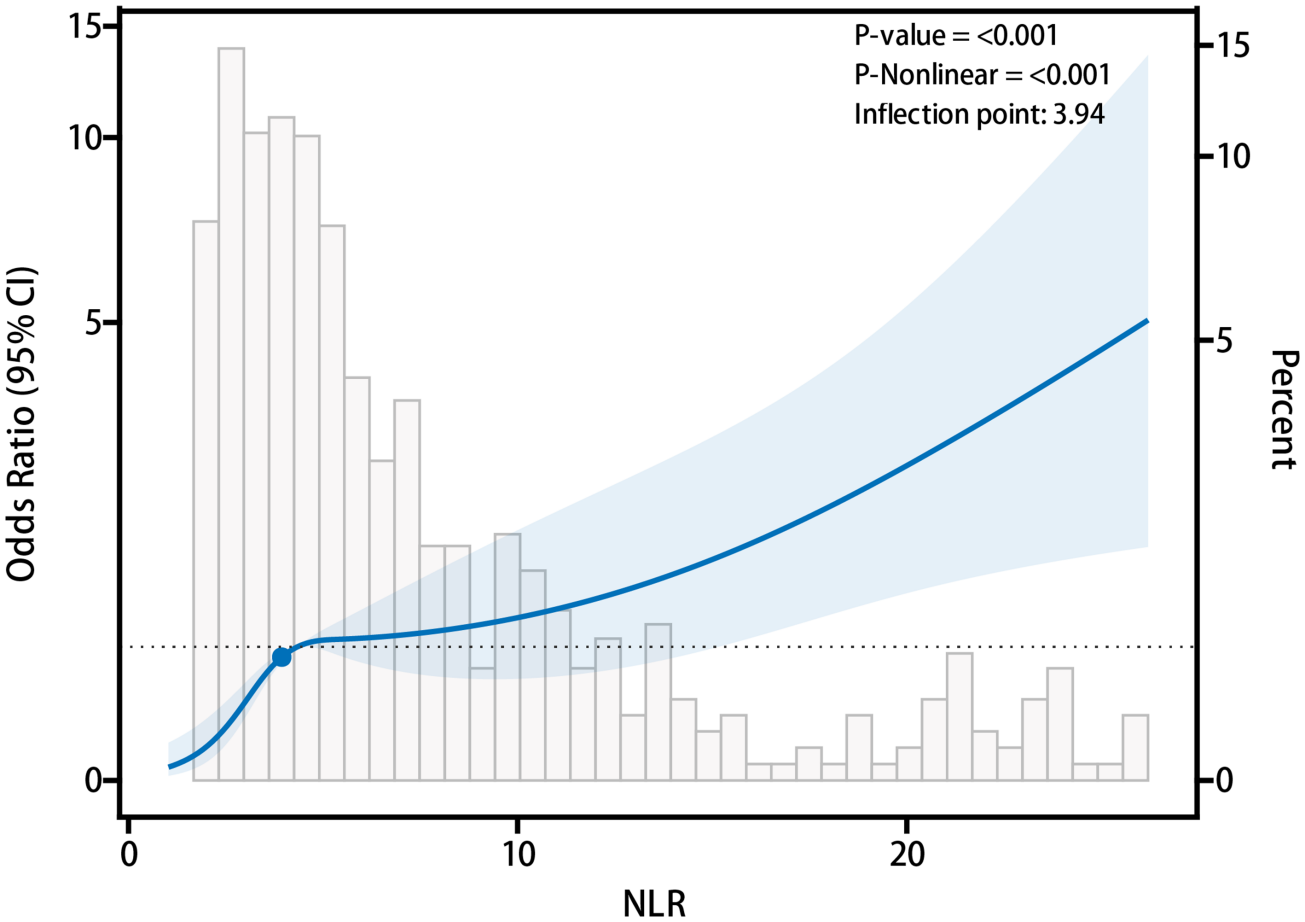
Nonlinear relationship between neutrophil to lymphocyte ratio (NLR) and post-chemotherapy pulmonary infection (PCPI).

After controlling for multiple confounding variables, including gender, age, alcohol consumption, smoking, diabetes, hypertension, heart disease, surgery, radiotherapy, body mass index, chemotherapy cycles, hospitalizations, and pleural effusion, the RCS analysis demonstrated a distinct “L”-shaped association between NLR and the incidence of PCPI (Figure 3). Specifically, a negative correlation with the risk of PCPI was observed at lower NLR values. Conversely, when the NLR value surpassed a certain threshold, the association with PCPI risk became positively pronounced. Further analysis pinpointed the inflection point of this nonlinear relationship at an NLR of 3.94. This indicates that NLR exerts minimal influence on the risk of PCPI below this value, whereas, above this threshold, an increase in NLR significantly heightens the risk. These findings suggest that while NLR may have a limited predictive capacity for PCPI at lower levels, its predictive capability is markedly enhanced when the value exceeds 3.94.

### 3.5 Subgroup analysis results

**Figure 4:**
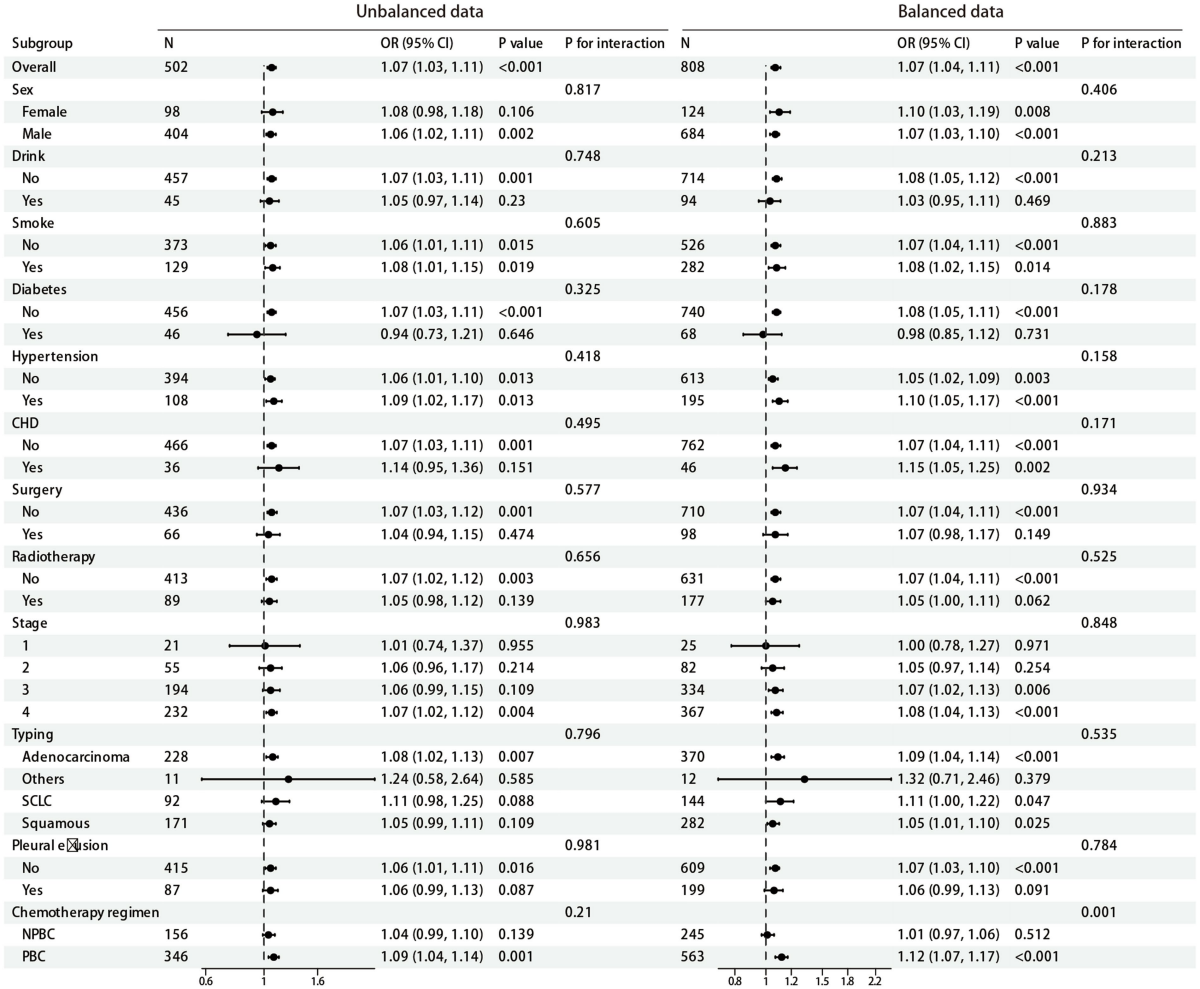
Forest map for subgroup analysis.

In the subgroup analysis, we investigated the association between NLR and PCPI across various subgroups within balanced and unbalanced datasets. We assessed the potential impact of factors such as gender, alcohol consumption, smoking, diabetes, hypertension, coronary heart disease, surgery, radiotherapy, tumor stage, tumor type, and pleural effusion using a univariate logistic regression model, with a particular focus on the significance of interaction effects (*p* for interaction). As illustrated in Figure 4, no significant differences were identified in the relationship between NLR and post-chemotherapy lung infection across different subgroups in unbalanced dataset, as all *p* for interaction values exceeded 0.05. Notably, while the relationship between NLR and post-chemotherapy lung infection showed statistical significance in subgroups defined by gender, smoking status, diabetes, and hypertension (*p*-values <0.05), the interaction effects of these factors did not achieve statistical significance (*p* for interaction >0.05). However, in the balanced dataset, within the chemotherapy regimen subgroups, a significant correlation was observed between NLR and outcomes for patients receiving the PBC regimen (OR = 1.12, 95% CI: 1.07–1.17, *p* < 0.001), while no significant association was noted for patients on the NPBC regimen (*p* = 0.512). Additionally, there was a significant interaction between the chemotherapy regimen subgroups (*p* for interaction = 0.001). No significant interactions were observed in other subgroups apart from the chemotherapy regimen (*p* for interaction >0.05).

### 3.6 Sensitivity analysis

In the sensitivity analysis, we assessed the robustness of the association between NLR and PCPI across various model configurations. Patient data with missing values were excluded from the analysis. Eighty-seven patients (17.33%) were omitted in the unbalanced dataset, whereas 122 patients (15.10%) were excluded in the balanced dataset. The analysis was performed separately on the balanced and unbalanced datasets, employing four distinct models (Model 1 to Model 4) to corroborate further the relationship between NLR and the risk of PCPI. The findings, as detailed in Table 3, indicate that within the unbalanced dataset, the analysis of continuous NLR variables revealed a significant positive correlation between NLR and PCPI, with OR ranging from 1.07 to 1.08 and all *p*-values less than 0.05. Similarly, the analysis of standardized NLR demonstrated a significant association with pulmonary infection risk, with ORs ranging from 1.51 to 1.61 and all *p*-values below 0.05. Quantile analysis further identified that the Q4 group, representing higher NLR values, showed a significantly elevated risk across multiple models, with ORs ranging from 1.90 to 2.34 and *p*-values spanning 0.019 to 0.097. The trend test corroborated the increasing NLR levels associated with a heightened risk of PCPI, with *p*-values for trends ranging from 0.010 to 0.059. In the balanced dataset, the analysis of continuous NLR variables also demonstrated a robust positive correlation, with ORs between 1.08 and 1.11 and all *p*-values less than 0.001. The results from the standardized NLR analysis further substantiated this correlation, with ORs ranging from 1.70 to 2.10 and all *p*-values below 0.001. In the quantile analysis, NLR values in the Q4 group were significantly associated with the risk of PCPI (OR range: 3.93–5.66, all *p*-values <0.001). The trend test similarly indicated that higher NLR levels corresponded to a greater risk of PCPI (*p* for trend <0.001). The sensitivity analysis results suggest that the association between NLR and PCPI remains stable across different models and quantile analyses, further supporting the reliability of NLR as a predictor, both in balanced and unbalanced datasets.

**Table 3:** Sensitivity analysis of the association between NLR and PCPI.

Variables	Model 1		Model 2		Model 3		Model 4	
	OR (95% CI)^1^	*p*	OR (95% CI)^1^	*p*	OR (95% CI)^1^	*p*	OR (95% CI)^1^	*p*
Unbalanced data								
NLR (continuous)	1.08 (1.03–1.12)	<0.001	1.07 (1.03–1.11)	0.002	1.07 (1.02–1.11)	0.004	1.07 (1.02–1.13)	0.009
NLR (standardized)	1.61 (1.24–2.08)	<0.001	1.54 (1.18–2.01)	0.002	1.51 (1.14–1.99)	0.004	1.59 (1.12–2.25)	0.009
NLR								
Q1	—		—		—		—	
Q2	0.92 (0.41–2.06)	0.836	0.90 (0.39–2.08)	0.805	0.85 (0.36–1.99)	0.708	0.73 (0.28–1.94)	0.535
Q3	1.23 (0.57–2.64)	0.599	1.06 (0.47–2.39)	0.884	1.07 (0.47–2.41)	0.876	1.10 (0.42–2.87)	0.850
Q4	2.34 (1.15–4.77)	0.019	2.07 (0.98–4.36)	0.056	1.90 (0.89–4.06)	0.097	2.28 (0.90–5.74)	0.081
*p* for trend		0.010		0.036		0.059		0.040
Balanced data								
NLR (continuous)	1.09 (1.06–1.13)	<0.001	1.08 (1.04–1.12)	<0.001	1.08 (1.04–1.12)	<0.001	1.11 (1.07–1.16)	<0.001
NLR (standardized)	1.87 (1.47–2.37)	<0.001	1.70 (1.34–2.16)	<0.001	1.72 (1.35–2.20)	<0.001	2.10 (1.57–2.79)	<0.001
NLR								
Q1	—		—		—		—	
Q2	2.23 (1.43–3.46)	<0.001	2.46 (1.54–3.95)	<0.001	2.44 (1.52–3.91)	<0.001	2.41 (1.34–4.31)	0.003
Q3	2.28 (1.46–3.55)	<0.001	1.91 (1.19–3.07)	0.008	1.91 (1.18–3.07)	0.008	2.21 (1.21–4.02)	0.010
Q4	4.78 (3.03–7.53)	<0.001	3.98 (2.46–6.42)	<0.001	3.93 (2.42–6.40)	<0.001	5.66 (3.07–10.44)	<0.001
*p* for trend		<0.001		<0.001		<0.001		<0.001

1 OR, odds ratio; CI, confidence interval. Model 1: No covariates were adjusted. Model 2: Adjusted for sex, age, drink, smoke, and BMI. Model 3: Adjusted for sex, age, drink, smoke, diabetes, hypertension, CHD, and BMI. Model 4: Adjusted for sex, age, drink, smoke, diabetes, hypertension, CHD, surgery, radiotherapy, BMI, stage, typing, chemotherapy cycle, number of hospitalizations, pleural effusion, and chemotherapy regimen.

## 4 Discussion

The objective of the present study was to assess the independent diagnostic significance of NLR in forecasting PCPI among patients with lung cancer. An analysis of clinical data from 502 lung cancer patients demonstrated that NLR functions as an independent risk factor for pulmonary infection after chemotherapy, applicable to both balanced and unbalanced datasets. Further examination indicated a significant increase in the risk of PCPI with elevated NLR values, revealing a distinct nonlinear relationship between NLR and PCPI. Subgroup and sensitivity analyses corroborated the robustness of these findings.

The findings of this study substantiate the critical role of NLR in the incidence of PCPI among patients with lung cancer. Our analysis identified NLR as an independent risk factor for such infections, irrespective of dataset balance. Specifically, within the balanced dataset, the OR for NLR was 1.12 (95% CI: 1.08–1.16), indicating that each unit increase in NLR significantly elevates the risk of pulmonary infection after chemotherapy. Furthermore, quartile analysis of NLR revealed that patients in the highest quartile (Q4) exhibited a markedly increased risk of after chemotherapy, with an OR of 5.64 (95% CI: 3.17–10.01, *p* < 0.001). These results are consistent with existing literature, highlighting the prognostic value of NLR as an indicator of the body’s inflammatory response in various tumors and infectious diseases (22–24). In lung cancer, chemotherapy-induced immunosuppression mainly affects the quantity and function of neutrophils and lymphocytes (25, 26). An elevated NLR indicates immune dysfunction and heightened systemic inflammation, which may contribute to the pathogenesis of pulmonary infections. Previous studies have shown that chemotherapy-induced immunosuppression increases lung cancer patients’ susceptibility to bacterial and fungal infections, and NLR, as a marker of inflammation, effectively reflects this immune dysfunction (27, 28). Therefore, NLR serves as an early screening indicator and provides clinicians with crucial information about patients’ immune status, aiding in predicting and preventing pulmonary infections.

Utilizing the RCS analysis, we examined the potential non-linear relationship between NLR and PCPI. The findings reveal that the association is not merely linear but exhibits a marked “L”-shaped pattern, indicating that the risk of PCPI remains relatively stable at lower NLR levels but increases sharply beyond a specific threshold. In this study, the inflection point was identified at NLR = 3.94, suggesting a critical threshold beyond which infection susceptibility markedly escalates. These results are consistent with previous studies, which propose that the influence of NLR is modulated by various factors, including the patient’s immune status, cancer stage, and chemotherapy regimen (12, 29). This may be because NLR reflects both systemic inflammation and immune status, and its impact may vary depending on its level. At lower NLR levels, it indicates that the immune system remains relatively intact, and slight changes in NLR have minimal impact on infection risk. When NLR is moderately elevated, the inflammatory response may still be compensatory, and the immune system may be able to effectively control infection risk. However, at higher NLR levels, the combination of excessive neutrophil-driven inflammation and suppressed lymphocyte-mediated adaptive immunity may lead to a state of immune dysfunction, increasing susceptibility to PCPI. This may explain why the risk of PCPI rises sharply when NLR exceeds a certain threshold (for example, NLR >3.94 in this study). From a clinical perspective, this threshold suggests that patients with an NLR below 3.94 may maintain a relatively stable immune response, whereas patients exceeding this level exhibit signs of immune dysregulation characterized by neutrophil-driven excessive inflammation and impaired lymphocyte-mediated adaptive immunity. When NLR is less than 3.94, the immune system remains functionally intact, and slight variations in NLR may not significantly affect infection risk. However, when NLR is greater than or equal to 3.94, the rapid escalation of PCPI risk can be attributed to a shift in systemic immune dysfunction. Here, excessive neutrophil activation leads to a pronounced inflammatory response, compromising lung epithelial integrity and increasing susceptibility to opportunistic infections. Simultaneously, lymphocytopenia impairs adaptive immunity, reducing the ability to effectively clear infections. The NLR threshold of 3.94 can serve as an early risk stratification marker to identify lung cancer patients at increased risk of PCPI after chemotherapy. Patients surpassing this threshold may benefit from closer infection monitoring, early preventive antibacterial strategies, and tailored supportive nursing interventions to mitigate infection-related complications. If future prospective studies validate these findings, this threshold could be incorporated into clinical decision algorithms, optimizing personalized infection prevention strategies. Furthermore, chemotherapy itself may have a dual effect on the immune system—initially causing temporary changes in NLR, but leading to long-term immunosuppression at higher NLR levels, further amplifying the risk of infection.

NLR has shown considerable potential in diagnosing and prognostic assessing various diseases, as documented in the existing literature. Among patients undergoing surgical interventions, NLR is correlated with surgical outcomes and postoperative complications. For instance, individuals with an NLR ≥2.7 demonstrate a significantly elevated risk of reduced recurrence-free survival (30). In oncological research, NLR, serving as an indicator of systemic inflammatory response, has been extensively investigated and found to be closely associated with the prognosis of patients across various cancer types. For example, in a study involving individuals with metastatic renal cell carcinoma, pre-treatment NLR was significantly associated with both progression-free survival (PFS) and overall survival (OS). The results indicated that patients with lower NLR exhibited a more favorable survival prognosis following sunitinib treatment (31). Additionally, in breast cancer patients, an increase in NLR post-radiotherapy is regarded as an adverse prognostic factor. Research has demonstrated a significant association between elevated NLR induced by radiotherapy and increased tumor recurrence rates, indicating that alterations in NLR may serve as a critical indicator for assessing the efficacy of radiotherapy (32). In investigations concerning colorectal cancer, the systemic inflammatory response is acknowledged as a marker of poor preoperative prognosis. Evidence suggests that systemic inflammation markers, including elevated levels of proinflammatory cytokines and acute-phase proteins, may contribute to cancer-related cachexia and unfavorable survival outcomes (33). Furthermore, studies have identified NLR as a potential prognostic biomarker that is strongly correlated with poor prognosis in patients with renal cell carcinoma. By integrating NLR with other established prognostic indicators, such as TNM staging and Fuhrman nuclear grading, a more precise prediction of patient prognosis can be achieved (34).

In lung cancer, NLR has been extensively employed as a prognostic marker, demonstrating its potential utility in chemotherapy and immunotherapy for lung cancer patients. Empirical evidence suggests that NLR holds substantial prognostic significance for individuals with non-small cell lung cancer (NSCLC) undergoing immunotherapy. Specifically, an elevated NLR is correlated with diminished progression-free survival (PFS) and overall survival (OS), particularly among patients receiving immune checkpoint inhibitors (ICIs) (35). Moreover, NLR has been utilized to prognosticate outcomes in advanced NSCLC patients undergoing chemotherapy, with pretreatment levels serving as predictors of survival outcomes (36). Additional research has investigated the synergistic use of NLR alongside other inflammatory markers. For example, the F-NLR score, which combines fibrinogen and NLR, has demonstrated prognostic predictive value in patients with resectable NSCLC. Those classified within the high-risk F-NLR group may derive more significant benefits from postoperative adjuvant therapy (37). NLR serves as an indicator reflecting systemic inflammation and immune status. An elevated NLR signifies both the intensification of neutrophil-driven inflammation and the suppression of lymphocyte-mediated immune responses (38). Chemotherapy-induced immunosuppression may further compromise host defense capabilities, thereby increasing susceptibility to infections after chemotherapy. Neutrophils release proinflammatory cytokines (such as IL-6 and TNF-α), which can potentially damage lung epithelial integrity and exacerbate the risk of infection (39, 40). Concurrently, lymphopenia indicates impaired adaptive immunity, diminishing the body’s ability to effectively clear infections. Studies have suggested that chemotherapy-induced immune dysfunction may elevate infection risk. For instance, a study on neutropenia highlighted that cancer patients experiencing chemotherapy-induced neutropenia face an increased risk of infectious complications (41). In this study, researchers emphasized that during neutropenia, the first instance of fever is often bacterial in origin, while subsequent fevers are primarily fungal. Therefore, the proper management of pulmonary infection complications following chemotherapy in lung cancer patients requires a profound understanding of the local epidemiology of infections, pathogenic microorganisms, and their drug resistance profiles. The findings of this study indicate that NLR possesses prognostic significance not only as a standalone marker but also in conjunction with other indicators to enhance predictive accuracy for the prognosis of lung cancer patients. This research further broadens the application of NLR by examining its role in secondary pulmonary infections following chemotherapy in lung cancer patients, thereby addressing a previously unexplored area of research. Unlike previous studies, this investigation utilizes various statistical analysis techniques, including RCS analysis and subgroup analysis, to affirm NLR’s independent diagnostic value and stability across diverse clinical contexts. Consequently, this study provides more robust evidence to inform clinical practice.

Despite observing some correlation between increased NLR levels and the occurrence of PCPI in lung cancer patients in this exploratory study, it is important to recognize the existence of several limitations. Firstly, this study was subject to the inherent limitations of a single-center retrospective design, which may affect the generalizability of our findings. Since all participants were recruited from the Central Hospital of Shaoyang, institutional and regional variations in patient characteristics, infection control practices, and chemotherapy regimens may exist, limiting the applicability of our results to a broader population. Additionally, retrospective data collection is prone to selection bias due to the potential for inadequate representation of certain patient subgroups. While we attempted to mitigate these biases through rigorous statistical analysis, including multivariable adjustment and sensitivity analysis, the possibility of residual confounding cannot be completely excluded. Secondly, despite adjusting for key covariates in our analysis, there may still exist certain potentially relevant confounding factors that are not present in our dataset. For instance, during the follow-up process, we did not collect other profound clinical variables, such as the performance status of patients and detailed smoking history (pack-years). These factors could potentially influence susceptibility to infection and immune function, and should be taken into account in future studies to improve the predictive accuracy of NLR for PCPI risk. Lastly, NLR was utilized as a solitary indicator without integration with other immune markers, such as WBC, CRP, or PCT. Future research should consider incorporating multiple inflammatory markers to enhance diagnostic sensitivity and specificity.

Future research should endeavor to validate the findings of this study within a larger, multicenter, prospective cohort and further investigate the diagnostic utility of integrating NLR with other immune markers. Additionally, employing molecular biology techniques to examine the mechanisms underlying alterations in NLR may elucidate the molecular basis of immunosuppression and PCPI in lung cancer patients. These investigations could establish NLR as a routine monitoring indicator for PCPI in lung cancer patients, thereby offering robust support for clinical decision-making.

## 5 Conclusion

This single-center retrospective study provides preliminary evidence suggesting that elevated NLR levels may serve as a potential independent predictor associated with PCPI development in lung cancer patients. While these findings tentatively support the diagnostic value of NLR in monitoring post-chemotherapy immune status, we emphasize that the observational nature and single-center design necessitate cautious interpretation. The results should be validated through prospective, multicenter studies with larger cohorts before clinical application. This exploratory analysis lays preliminary groundwork for future investigations examining the integration of NLR with multidimensional biomarkers to develop more robust predictive models for PCPI risk stratification.

## Data Availability

The raw data supporting the conclusions of this article will be made available by the authors, without undue reservation.

## Supplementary material

SUPPLEMENTARY FIGURE 1 Lollipop chart of missing rates for study variables.

SUPPLEMENTARY FIGURE 2 Forest plot for univariate and multivariate regression analysis of unbalanced dataset.

## References

[1] Luo G Zhang Y Etxeberria J Arnold M Cai X Hao Y. Projections of lung cancer incidence by 2035 in 40 countries worldwide: population-based study. JMIR Public Health Surveill. (2023) 9:e43651. doi: 10.2196/43651, PMID: 36800235 PMC9984998

[2] Li C Lei S Ding L Xu Y Wu X Wang H. Global burden and trends of lung cancer incidence and mortality. Chin Med J. (2023) 136:1583–90. doi: 10.1097/cm9.0000000000002529, PMID: 37027426 PMC10325747

[3] Cai Y Xu Y Xu D Wang Y Wang X Sun C. Considerations in treating patients with advance lung cancer during the epidemic outbreak of novel coronavirus (SARS-CoV-2). Med Oncol. (2020) 37:78. doi: 10.1007/s12032-020-01401-w, PMID: 32748212 PMC7396726

[4] Wisnivesky JP Smith CB Packer S Strauss GM Lurslurchachai L Federman A. Survival and risk of adverse events in older patients receiving postoperative adjuvant chemotherapy for resected stages II-IIIA lung cancer: observational cohort study. BMJ. (2011) 343:d4013. doi: 10.1136/bmj.d4013, PMID: 21757436 PMC3136092

[5] Coventry BJ Ashdown ML. Complete clinical responses to cancer therapy caused by multiple divergent approaches: a repeating theme lost in translation. Cancer Manag Res. (2012) 4:137–49. doi: 10.2147/cmar.S31887, PMID: 22740774 PMC3379856

[6] Lin YJ Wei KC Chen PY Lim M Hwang TL. Roles of neutrophils in glioma and brain metastases. Front Immunol. (2021) 12:701383. doi: 10.3389/fimmu.2021.701383, PMID: 34484197 PMC8411705

[7] van Luijk IF Smith SM Marte Ojeda MC Oei AL Kenter GG Jordanova ES. A review of the effects of cervical cancer standard treatment on immune parameters in peripheral blood, tumor draining lymph nodes, and local tumor microenvironment. J Clin Med. (2022) 11. doi: 10.3390/jcm11092277, PMID: 35566403 PMC9102821

[8] Kaplan MJ. Role of neutrophils in systemic autoimmune diseases. Arthritis Res Ther. (2013) 15:219. doi: 10.1186/ar4325, PMID: 24286137 PMC3978765

[9] Delgado A Guddati AK. Infections in hospitalized cancer patients. World J Oncol. (2021) 12:195–205. doi: 10.14740/wjon1410, PMID: 35059079 PMC8734501

[10] Sierra-Rodero B Cruz-Bermúdez A Nadal E Garitaonaindía Y Insa A Mosquera J. Clinical and molecular parameters associated to pneumonitis development in non-small-cell lung cancer patients receiving chemoimmunotherapy from NADIM trial. J Immunother Cancer. (2021) 9:e002804. doi: 10.1136/jitc-2021-002804, PMID: 34446577 PMC8395363

[11] Long K Suresh K. Pulmonary toxicity of systemic lung cancer therapy. Respirology. (2020) 25:72–9. doi: 10.1111/resp.13915, PMID: 32729207

[12] Russell CD Parajuli A Gale HJ Bulteel NS Schuetz P de Jager CPC. The utility of peripheral blood leucocyte ratios as biomarkers in infectious diseases: a systematic review and meta-analysis. J Infect. (2019) 78:339–48. doi: 10.1016/j.jinf.2019.02.006, PMID: 30802469 PMC7173077

[13] Nagy GR Kemény L Bata-Csörgő Z. Neutrophil-to-lymphocyte ratio: a biomarker for predicting systemic involvement in adult IgA vasculitis patients. J Eur Acad Dermatol Venereol. (2017) 31:1033–7. doi: 10.1111/jdv.14176, PMID: 28222228

[14] Stotz M Gerger A Eisner F Szkandera J Loibner H Ress AL. Increased neutrophil-lymphocyte ratio is a poor prognostic factor in patients with primary operable and inoperable pancreatic cancer. Br J Cancer. (2013) 109:416–21. doi: 10.1038/bjc.2013.332, PMID: 23799847 PMC3721392

[15] Zinellu A Zinellu E Mangoni AA Pau MC Carru C Pirina P. Clinical significance of the neutrophil-to-lymphocyte ratio and platelet-to-lymphocyte ratio in acute exacerbations of COPD: present and future. Eur Respir Rev. (2022) 31. doi: 10.1183/16000617.0095-2022, PMID: 36323421 PMC9724880

[16] Teperman J Carruthers D Guo Y Barnett MP Harris AA Sedlis SP. Relationship between neutrophil-lymphocyte ratio and severity of lower extremity peripheral artery disease. Int J Cardiol. (2017) 228:201–4. doi: 10.1016/j.ijcard.2016.11.097, PMID: 27865186 PMC8464161

[17] Moisa E Corneci D Negoita S Filimon CR Serbu A Negutu MI. Dynamic changes of the neutrophil-to-lymphocyte ratio, systemic inflammation index, and derived neutrophil-to-lymphocyte ratio independently predict invasive mechanical ventilation need and death in critically ill COVID-19 patients. Biomedicines. (2021) 9:1656. doi: 10.3390/biomedicines9111656, PMID: 34829883 PMC8615772

[18] Zitvogel L Kepp O Kroemer G. Immune parameters affecting the efficacy of chemotherapeutic regimens. Nat Rev Clin Oncol. (2011) 8:151–60. doi: 10.1038/nrclinonc.2010.223, PMID: 21364688

[19] Donskov F. Immunomonitoring and prognostic relevance of neutrophils in clinical trials. Semin Cancer Biol. (2013) 23:200–7. doi: 10.1016/j.semcancer.2013.02.001, PMID: 23403174

[20] Öhrmalm L Smedman C Wong M Broliden K Tolfvenstam T Norbeck O. Decreased functional T lymphocyte-mediated cytokine responses in patients with chemotherapy-induced neutropenia. J Intern Med. (2013) 274:363–70. doi: 10.1111/joim.12100, PMID: 23789642

[21] Xu Z Shen D Kou Y Nie T. A synthetic minority oversampling technique based on Gaussian mixture model filtering for imbalanced data classification. IEEE Trans Neural Netw Learn Syst. (2024) 35:3740–53. doi: 10.1109/tnnls.2022.3197156, PMID: 35984792

[22] Wang S Dong L Pei G Jiang Z Qin A Tan J. High neutrophil-to-lymphocyte ratio is an independent risk factor for end stage renal diseases in IgA nephropathy. Front Immunol. (2021) 12:700224. doi: 10.3389/fimmu.2021.700224, PMID: 34456912 PMC8387559

[23] Song Q Xu SX Wu JZ Ling L Wang S Shu XH. The preoperative platelet to neutrophil ratio and lymphocyte to monocyte ratio are superior prognostic indicators compared with other inflammatory biomarkers in ovarian cancer. Front Immunol. (2023) 14:1177403. doi: 10.3389/fimmu.2023.1177403, PMID: 37457691 PMC10347525

[24] Naess A Nilssen SS Mo R Eide GE Sjursen H. Role of neutrophil to lymphocyte and monocyte to lymphocyte ratios in the diagnosis of bacterial infection in patients with fever. Infection. (2017) 45:299–307. doi: 10.1007/s15010-016-0972-1, PMID: 27995553 PMC5488068

[25] Carus A Gurney H Gebski V Harnett P Hui R Kefford R. Impact of baseline and nadir neutrophil index in non-small cell lung cancer and ovarian cancer patients: assessment of chemotherapy for resolution of unfavourable neutrophilia. J Transl Med. (2013) 11:189. doi: 10.1186/1479-5876-11-189, PMID: 23945200 PMC3751486

[26] Ocana A Nieto-Jiménez C Pandiella A Templeton AJ. Neutrophils in cancer: prognostic role and therapeutic strategies. Mol Cancer. (2017) 16:137. doi: 10.1186/s12943-017-0707-7, PMID: 28810877 PMC5558711

[27] Wiegand SB Paal M Jung J Guba M Lange CM Schneider C. Importance of the neutrophil-to-lymphocyte ratio as a marker for microbiological specimens in critically ill patients after liver or lung transplantation. Infection. (2024). doi: 10.1007/s15010-024-02398-4, PMID: 39586958 PMC11971184

[28] Bartlett EK Flynn JR Panageas KS Ferraro RA Sta Cruz JM Postow MA. High neutrophil-to-lymphocyte ratio (NLR) is associated with treatment failure and death in patients who have melanoma treated with PD-1 inhibitor monotherapy. Cancer. (2020) 126:76–85. doi: 10.1002/cncr.32506, PMID: 31584709 PMC6906249

[29] Curbelo J Rajas O Arnalich B Galván-Román JM Luquero-Bueno S Ortega-Gómez M. Neutrophil count percentage and neutrophil-lymphocyte ratio as prognostic markers in patients hospitalized for community-acquired pneumonia. Arch Bronconeumol. (2019) 55:472–7. doi: 10.1016/j.arbres.2019.02.005, PMID: 30914210

[30] Allenet C Klein C Rouget B Margue G Capon G Alezra E. Can pre-operative neutrophil-to-lymphocyte ratio (NLR) help predict non-metastatic renal carcinoma recurrence after nephrectomy? (UroCCR-61 study). Cancers. (2022) 14:5692. doi: 10.3390/cancers14225692, PMID: 36428784 PMC9688470

[31] Keizman D Ish-Shalom M Huang P Eisenberger MA Pili R Hammers H. The association of pre-treatment neutrophil to lymphocyte ratio with response rate, progression free survival and overall survival of patients treated with sunitinib for metastatic renal cell carcinoma. Eur J Cancer. (2012) 48:202:208. doi: 10.1016/j.ejca.2011.09.001 22018713 PMC3483077

[32] Yoon CI Kim D Ahn SG Bae SJ Cha C Park S. Radiotherapy-induced high neutrophil-to-lymphocyte ratio is a negative prognostic factor in patients with breast cancer. Cancers. (2020) 12:1896. doi: 10.3390/cancers12071896, PMID: 32674376 PMC7409084

[33] Tuomisto AE Mäkinen MJ Väyrynen JP. Systemic inflammation in colorectal cancer: underlying factors, effects, and prognostic significance. World J Gastroenterol. (2019) 25:4383–404. doi: 10.3748/wjg.v25.i31.4383, PMID: 31496619 PMC6710177

[34] Shao Y Wu B Jia W Zhang Z Chen Q Wang D. Prognostic value of pretreatment neutrophil-to-lymphocyte ratio in renal cell carcinoma: a systematic review and meta-analysis. BMC Urol. (2020) 20:90. doi: 10.1186/s12894-020-00665-8, PMID: 32631294 PMC7339475

[35] Bryant AK Sankar K Strohbehn GW Zhao L Elliott D Qin A. Prognostic and predictive value of neutrophil-to-lymphocyte ratio with adjuvant immunotherapy in stage III non-small-cell lung cancer. Lung Cancer. (2022) 163:35–41. doi: 10.1016/j.lungcan.2021.11.021, PMID: 34896803 PMC8770596

[36] Mandaliya H Jones M Oldmeadow C Nordman II. Prognostic biomarkers in stage IV non-small cell lung cancer (NSCLC): neutrophil to lymphocyte ratio (NLR), lymphocyte to monocyte ratio (LMR), platelet to lymphocyte ratio (PLR) and advanced lung cancer inflammation index (ALI). Transl Lung Cancer Res. (2019) 8:886–94. doi: 10.21037/tlcr.2019.11.16, PMID: 32010567 PMC6976360

[37] Wang H Zhao J Zhang M Han L Wang M Xingde L. The combination of plasma fibrinogen and neutrophil lymphocyte ratio (F-NLR) is a predictive factor in patients with resectable non small cell lung cancer. J Cell Physiol. (2018) 233:4216–24. doi: 10.1002/jcp.26239, PMID: 29057536

[38] Zahorec R. Neutrophil-to-lymphocyte ratio, past, present and future perspectives. Bratisl Lek Listy. (2021) 122:474–88. doi: 10.4149/bll_2021_078, PMID: 34161115

[39] Riedemann NC Guo RF Hollmann TJ Gao H Neff TA Reuben JS. Regulatory role of C5a in LPS-induced IL-6 production by neutrophils during sepsis. FASEB J. (2004) 18:370–2. doi: 10.1096/fj.03-0708fje, PMID: 14688199

[40] Zou S Jie H Han X Wang J. The role of neutrophil extracellular traps in sepsis and sepsis-related acute lung injury. Int Immunopharmacol. (2023) 124:110436. doi: 10.1016/j.intimp.2023.110436, PMID: 37688916

[41] Menichetti F. Infectious complications in neutropenic cancer patients. Intern Emerg Med. (2010) 5:21–5. doi: 10.1007/s11739-010-0456-8, PMID: 20865470

