# Supplementary figures and images for: The association of neutrophil-to-lymphocyte ratio with post-chemotherapy pulmonary infection in lung cancer patients
Source: Front Med (Lausanne). 2025 Apr 9;12:1559702. doi: 10.3389/fmed.2025.1559702 (PMC12014436; doi:10.3389/fmed.2025.1559702)

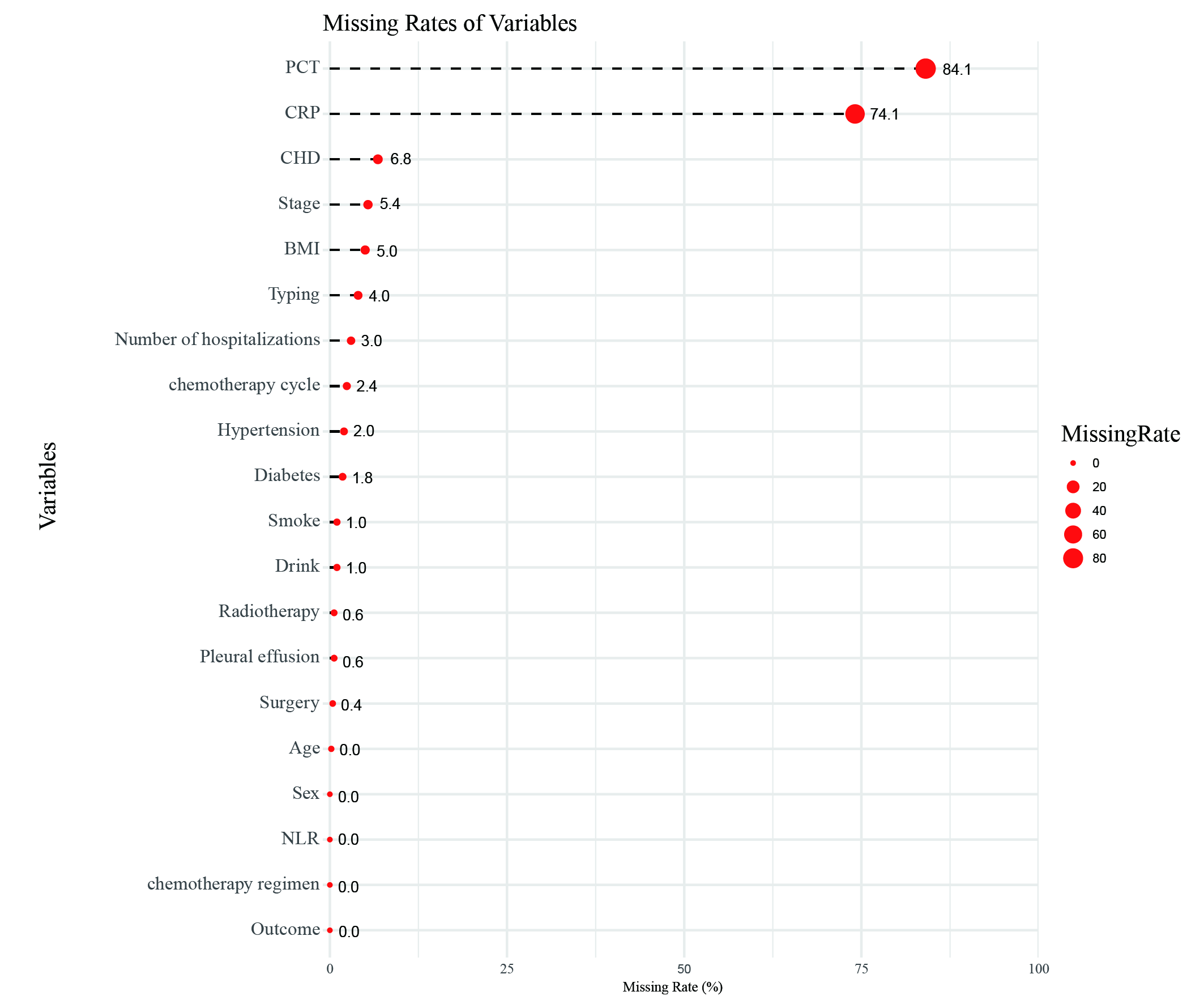

Supplement: SUPPLEMENTARY FIGURE 1 — Lollipop chart of missing rates for study variables. [file Image_1.TIF]

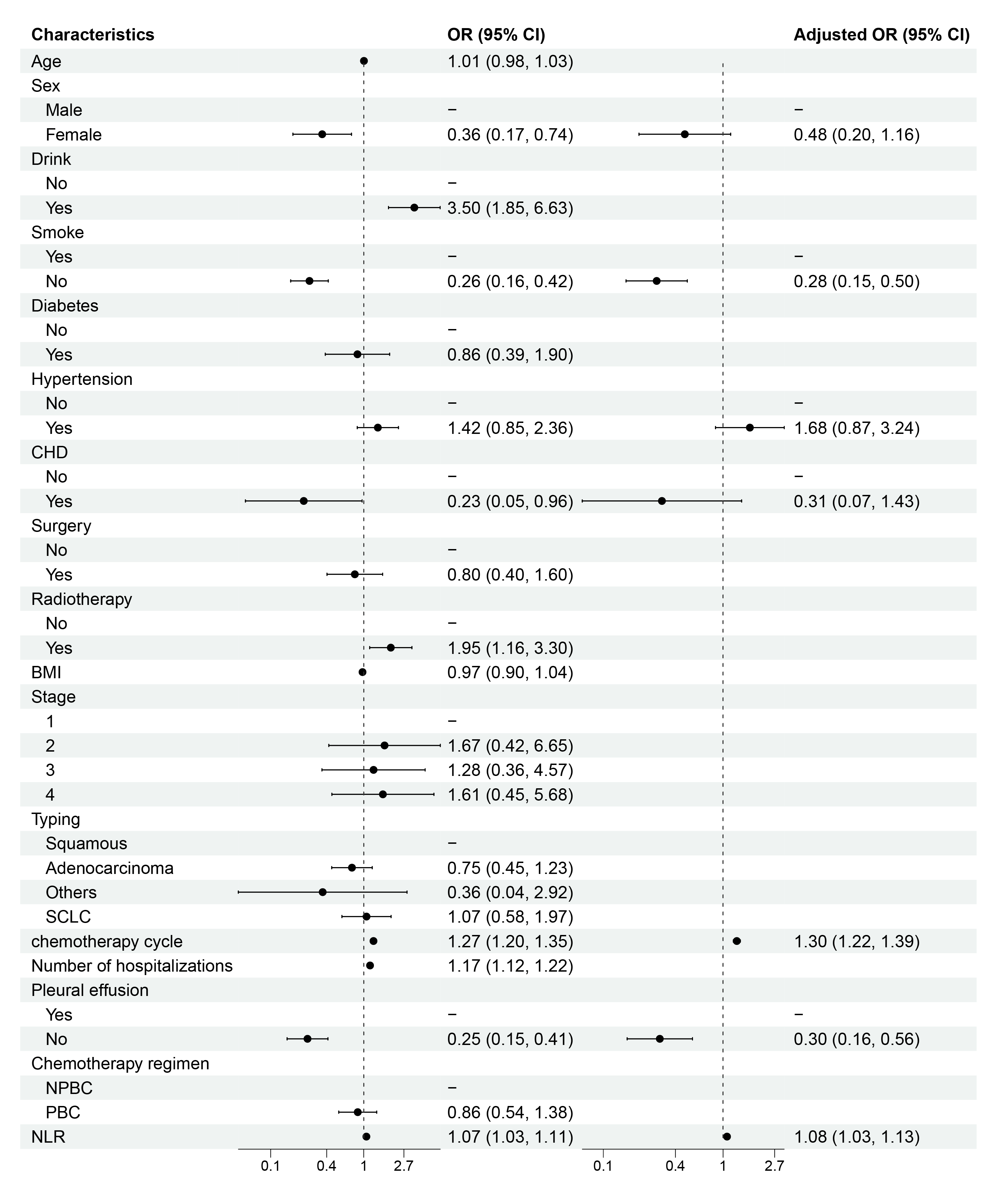

Supplement: SUPPLEMENTARY FIGURE 2 — Forest plot for univariate and multivariate regression analysis of unbalanced dataset. [file Image_2.TIF]
