# Supplementary material for: The association of neutrophil-to-lymphocyte ratio with post-chemotherapy pulmonary infection in lung cancer patients
Source: Front Med (Lausanne). 2025 Apr 9;12:1559702. doi: 10.3389/fmed.2025.1559702 (PMC12014436; doi:10.3389/fmed.2025.1559702)
Supplement: Supplementary file 3 [file Supplementary_file_1.docx]

Supplementary Table 1. Description of the study variables.

| **No** | **Variables** | **Description** | **Types** | **Values** |
| --- | --- | --- | --- | --- |
| 1 | Age | Age of the patient (years) | Continuous | 35-83 |
| 2 | NLR | Neutrophil-to-Lymphocyte Ratio | Continuous | 0.44-71.74 |
| 3 | CRP | C-reactive protein | Continuous | 0.182-301 |
| 4 | PCT | Procalcitonin | Continuous | 0.02-5.54 |
| 5 | BMI | Body mass index (kg/m2) | Continuous | 11.43-31.83 |
| 6 | chemotherapy cycles | Number of chemotherapy cycles | Continuous | 1-32 |
| 7 | Number of hospitalizations | Total number of hospitalizations after diagnosis of lung cancer | Continuous | 1-45 |
| 8 | Sex | Sex of the patient | Categorical | 1: male, 2: female |
| 9 | Surgery | Whether or not lung resection is performed | Categorical | 0: No, 1: Yes |
| 10 | Radiotherapy | History of radiation therapy | Categorical | 0: No, 1: Yes |
| 11 | Stage | Stages of Lung Cancer, Count(%) | Categorical | Ⅰstage, 4.18%; Ⅱ stage, 10.96%;Ⅲ stage, 38.65%;Ⅳ stage, 46.22%. |
| 12 | Typing | Lung Cancer Typing, Count(%) | Categorical | adenocarcinoma, 45.42%; squamous, 34.06%;  SCLC, 18.33%;  Others, 2.19% |
| 13 | Pleural effusion | Pleural effusion present | Categorical | 0: No, 1: Yes |
| 14 | Drink | Drinking History | Categorical | 0: No, 1: Yes |
| 15 | Smoke | Smoking History | Categorical | 0: No, 1: Yes |
| 16 | Diabetes | History of diabetes | Categorical | 0: No, 1: Yes |
| 17 | Hypertension | History of Hypertension | Categorical | 0: No, 1: Yes |
| 18 | CHD | History of coronary heart disease | Categorical | 0: No, 1: Yes |
| 19 | Chemotherapy regimens | Chemotherapy regimens | Categorical | 1: Platinum-based chemotherapy (PBC)  2: Non-platinum-based chemotherapy (NPBC) |
| 20 | Outcome | Post-chemotherapy pulmonary infection (PIPC) | Categorical | 0: No, 1: Yes |
