# Supplementary material for: The association of neutrophil-to-lymphocyte ratio with post-chemotherapy pulmonary infection in lung cancer patients
Source: Front Med (Lausanne). 2025 Apr 9;12:1559702. doi: 10.3389/fmed.2025.1559702 (PMC12014436; doi:10.3389/fmed.2025.1559702)
Supplement: Supplementary file 4 [file Supplementary_file_2.docx]

Supplementary Table 2. Baseline information table for unbalanced dataset.

| **Variables** | **Outcome** | | | P-Value |
| --- | --- | --- | --- | --- |
|  | Overall, N = 502^1^ | NPCPI, N = 404^1^ | PCPI, N = 98^1^ |  |
| **Age** | 65.00 (58.00, 71.00) | 65.00 (58.00, 71.00) | 65.00 (59.00, 69.00) | 0.875^2^ |
| **BMI** | 21.82 ± 3.15 | 21.88 ± 3.13 | 21.58 ± 3.21 | 0.415^3^ |
| **chemotherapy cycle** | 3.00 (1.00, 6.00) | 3.00 (1.00, 5.00) | 6.00 (4.00, 10.75) | <0.001^2^ |
| **Number of hospitalizations** | 5.00 (3.00, 8.00) | 4.00 (2.00, 7.00) | 10.00 (5.25, 15.00) | <0.001^2^ |
| **Sex** |  |  |  | 0.004^4^ |
| Female | 98 (19.52%) | 89 (22.03%) | 9 (9.18%) |  |
| Male | 404 (80.48%) | 315 (77.97%) | 89 (90.82%) |  |
| **Drink** |  |  |  | <0.001^4^ |
| No | 457 (91.04%) | 378 (93.56%) | 79 (80.61%) |  |
| Yes | 45 (8.96%) | 26 (6.44%) | 19 (19.39%) |  |
| **Smoke** |  |  |  | <0.001^4^ |
| No | 373 (74.30%) | 323 (79.95%) | 50 (51.02%) |  |
| Yes | 129 (25.70%) | 81 (20.05%) | 48 (48.98%) |  |
| **Diabetes** |  |  |  | 0.702^4^ |
| No | 456 (90.84%) | 366 (90.59%) | 90 (91.84%) |  |
| Yes | 46 (9.16%) | 38 (9.41%) | 8 (8.16%) |  |
| **Hypertension** |  |  |  | 0.178^4^ |
| No | 394 (78.49%) | 322 (79.70%) | 72 (73.47%) |  |
| Yes | 108 (21.51%) | 82 (20.30%) | 26 (26.53%) |  |
| **CHD** |  |  |  | 0.028^4^ |
| No | 466 (92.83%) | 370 (91.58%) | 96 (97.96%) |  |
| Yes | 36 (7.17%) | 34 (8.42%) | 2 (2.04%) |  |
| **Surgery** |  |  |  | 0.530^4^ |
| No | 436 (86.85%) | 349 (86.39%) | 87 (88.78%) |  |
| Yes | 66 (13.15%) | 55 (13.61%) | 11 (11.22%) |  |
| **Radiotherapy** |  |  |  | 0.011^4^ |
| No | 413 (82.27%) | 341 (84.41%) | 72 (73.47%) |  |
| Yes | 89 (17.73%) | 63 (15.59%) | 26 (26.53%) |  |
| **Stage** |  |  |  | 0.733^5^ |
| Ⅰ stage | 21 (4.18%) | 18 (4.46%) | 3 (3.06%) |  |
| Ⅱ stage | 55 (10.96%) | 43 (10.64%) | 12 (12.24%) |  |
| Ⅲ stage | 194 (38.65%) | 160 (39.60%) | 34 (34.69%) |  |
| Ⅳ stage | 232 (46.22%) | 183 (45.30%) | 49 (50.00%) |  |
| **Typing** |  |  |  | 0.485^5^ |
| Adenocarcinoma | 228 (45.42%) | 189 (46.78%) | 39 (39.80%) |  |
| Squamous | 171 (34.06%) | 134 (33.17%) | 37 (37.76%) |  |
| SCLC | 92 (18.33%) | 71 (17.57%) | 21 (21.43%) |  |
| Others | 11 (2.19%) | 10 (2.48%) | 1 (1.02%) |  |
| **Pleural effusion** |  |  |  | <0.001^4^ |
| No | 415 (82.67%) | 353 (87.38%) | 62 (63.27%) |  |
| Yes | 87 (17.33%) | 51 (12.62%) | 36 (36.73%) |  |
| **Chemotherapy regimen** |  |  |  | 0.536^4^ |
| PBC | 346 (68.92%) | 281 (69.55%) | 65 (66.33%) |  |
| NPBC | 156 (31.08%) | 123 (30.45%) | 33 (33.67%) |  |
| **NLR** | 3.91 (2.56, 6.33) | 3.73 (2.52, 6.00) | 4.93 (3.21, 9.75) | <0.001^2^ |

^1^Median (IQR); Mean ± SD; n (%), ^2^Wilcoxon rank sum test, ^3^Welch Two Sample t-test, ^4^Pearson's Chi-squared test, ^5^Fisher's exact test.
