# Supplementary material for: The association of neutrophil-to-lymphocyte ratio with post-chemotherapy pulmonary infection in lung cancer patients
Source: Front Med (Lausanne). 2025 Apr 9;12:1559702. doi: 10.3389/fmed.2025.1559702 (PMC12014436; doi:10.3389/fmed.2025.1559702)
Supplement: Supplementary file 5 [file Supplementary_file_3.docx]

Supplementary Table 3. Baseline Characteristics of Study Subjects by NLR Quartiles (Q1-Q4) for the balanced dataset.

| **Variables** | **NLR group** | | | | | **p-value** |
| --- | --- | --- | --- | --- | --- | --- |
|  | Overall, N = 808^1^ | Q1, N = 199^1^ | Q2, N = 205^1^ | Q3, N = 202^1^ | Q4, N = 202^1^ |  |
| **Age** | 65.00 (58.00, 70.00) | 64.00 (57.00, 69.00) | 63.00 (58.00, 71.00) | 65.00 (59.00, 71.00) | 66.00 (61.00, 69.00) | 0.184^2^ |
| **BMI** | 21.64 (19.87, 23.60) | 22.41 (20.97, 24.66) | 21.63 (19.89, 23.59) | 21.12 (19.05, 22.95) | 21.29 (19.74, 23.44) | <0.001^2^ |
| **chemotherapy cycle** | 4.00 (2.00, 8.00) | 4.00 (2.00, 7.00) | 4.00 (3.00, 10.00) | 4.00 (2.00, 9.00) | 4.00 (2.00, 6.00) | 0.007^2^ |
| **Number of hospitalizations** | 6.00 (3.00, 11.00) | 6.00 (3.00, 11.00) | 7.00 (4.00, 13.00) | 6.00 (4.00, 11.00) | 6.00 (3.00, 9.75) | 0.044^2^ |
| **Sex** |  |  |  |  |  | 0.029^3^ |
| Male | 684 (84.65%) | 167 (83.92%) | 164 (80.00%) | 170 (84.16%) | 183 (90.59%) |  |
| Female | 124 (15.35%) | 32 (16.08%) | 41 (20.00%) | 32 (15.84%) | 19 (9.41%) |  |
| **Drink** |  |  |  |  |  | 0.812^3^ |
| No | 714 (88.37%) | 177 (88.94%) | 181 (88.29%) | 175 (86.63%) | 181 (89.60%) |  |
| Yes | 94 (11.63%) | 22 (11.06%) | 24 (11.71%) | 27 (13.37%) | 21 (10.40%) |  |
| **Smoke** |  |  |  |  |  | 0.035^3^ |
| Yes | 282 (34.90%) | 59 (29.65%) | 65 (31.71%) | 86 (42.57%) | 72 (35.64%) |  |
| No | 526 (65.10%) | 140 (70.35%) | 140 (68.29%) | 116 (57.43%) | 130 (64.36%) |  |
| **Diabetes** |  |  |  |  |  | 0.280^3^ |
| No | 740 (91.58%) | 176 (88.44%) | 188 (91.71%) | 187 (92.57%) | 189 (93.56%) |  |
| Yes | 68 (8.42%) | 23 (11.56%) | 17 (8.29%) | 15 (7.43%) | 13 (6.44%) |  |
| **Hypertension** |  |  |  |  |  | <0.001^3^ |
| No | 613 (75.87%) | 161 (80.90%) | 164 (80.00%) | 161 (79.70%) | 127 (62.87%) |  |
| Yes | 195 (24.13%) | 38 (19.10%) | 41 (20.00%) | 41 (20.30%) | 75 (37.13%) |  |
| **CHD** |  |  |  |  |  | 0.481^3^ |
| No | 762 (94.31%) | 185 (92.96%) | 196 (95.61%) | 193 (95.54%) | 188 (93.07%) |  |
| Yes | 46 (5.69%) | 14 (7.04%) | 9 (4.39%) | 9 (4.46%) | 14 (6.93%) |  |
| **Surgery** |  |  |  |  |  | <0.001^3^ |
| No | 710 (87.87%) | 158 (79.40%) | 188 (91.71%) | 188 (93.07%) | 176 (87.13%) |  |
| Yes | 98 (12.13%) | 41 (20.60%) | 17 (8.29%) | 14 (6.93%) | 26 (12.87%) |  |
| **Radiotherapy** |  |  |  |  |  | <0.001^3^ |
| No | 631 (78.09%) | 172 (86.43%) | 170 (82.93%) | 149 (73.76%) | 140 (69.31%) |  |
| Yes | 177 (21.91%) | 27 (13.57%) | 35 (17.07%) | 53 (26.24%) | 62 (30.69%) |  |
| **Stage** |  |  |  |  |  | <0.001^3^ |
| Ⅰ stage | 25 (3.09%) | 14 (7.04%) | 4 (1.95%) | 3 (1.49%) | 4 (1.98%) |  |
| Ⅱ stage | 82 (10.15%) | 34 (17.09%) | 15 (7.32%) | 17 (8.42%) | 16 (7.92%) |  |
| Ⅲ stage | 334 (41.34%) | 83 (41.71%) | 90 (43.90%) | 90 (44.55%) | 71 (35.15%) |  |
| Ⅳ stage | 367 (45.42%) | 68 (34.17%) | 96 (46.83%) | 92 (45.54%) | 111 (54.95%) |  |
| **Pleural effusion** |  |  |  |  |  | <0.001^3^ |
| Yes | 199 (24.63%) | 22 (11.06%) | 48 (23.41%) | 58 (28.71%) | 71 (35.15%) |  |
| No | 609 (75.37%) | 177 (88.94%) | 157 (76.59%) | 144 (71.29%) | 131 (64.85%) |  |
| **Chemotherapy regimen** |  |  |  |  |  | 0.182^3^ |
| NPBC | 245 (30.32%) | 59 (29.65%) | 69 (33.66%) | 50 (24.75%) | 67 (33.17%) |  |
| PBC | 563 (69.68%) | 140 (70.35%) | 136 (66.34%) | 152 (75.25%) | 135 (66.83%) |  |

^1^Mean ± SD; n (%), ^2^One-way ANOVA, ^3^Pearson's Chi-squared test.
