# Supplementary material for: The association of neutrophil-to-lymphocyte ratio with post-chemotherapy pulmonary infection in lung cancer patients
Source: Front Med (Lausanne). 2025 Apr 9;12:1559702. doi: 10.3389/fmed.2025.1559702 (PMC12014436; doi:10.3389/fmed.2025.1559702)
Supplement: Supplementary file 6 [file Supplementary_file_4.docx]

Supplementary Table 4. Baseline Characteristics of Study Subjects by NLR Quartiles (Q1-Q4) for the unbalanced dataset.

| **Variables** | **NLR group** | | | | | **p-value** |
| --- | --- | --- | --- | --- | --- | --- |
|  | **Overall, N = 502^1^** | **Q1, N = 125^1^** | **Q2, N = 125^1^** | **Q3, N = 126^1^** | **Q4, N = 126^1^** |  |
| **Age** | 65.00 (58.00, 71.00) | 64.00 (57.00, 69.00) | 63.00 (57.00, 72.00) | 65.00 (59.00, 72.00) | 66.00 (59.00, 71.00) | 0.169^2^ |
| **BMI** | 21.82 ± 3.15 | 22.80 ± 3.10 | 22.03 ± 2.96 | 20.92 ± 3.04 | 21.54 ± 3.22 | <0.001^3^ |
| **chemotherapy cycle** | 3.00 (1.00, 6.00) | 3.00 (2.00, 6.00) | 3.00 (1.00, 5.00) | 3.00 (1.00, 6.00) | 3.00 (1.00, 6.00) | 0.731^2^ |
| **Number of hospitalizations** | 5.00 (3.00, 8.00) | 5.00 (3.00, 9.00) | 5.00 (3.00, 8.00) | 5.00 (3.00, 8.00) | 5.00 (2.00, 8.00) | 0.855^2^ |
| **Sex** |  |  |  |  |  | 0.201^4^ |
| Female | 98 (19.52%) | 25 (20.00%) | 30 (24.00%) | 26 (20.63%) | 17 (13.49%) |  |
| Male | 404 (80.48%) | 100 (80.00%) | 95 (76.00%) | 100 (79.37%) | 109 (86.51%) |  |
| **Drink** |  |  |  |  |  | 0.337^4^ |
| No | 457 (91.04%) | 111 (88.80%) | 118 (94.40%) | 116 (92.06%) | 112 (88.89%) |  |
| Yes | 45 (8.96%) | 14 (11.20%) | 7 (5.60%) | 10 (7.94%) | 14 (11.11%) |  |
| **Smoke** |  |  |  |  |  | 0.208^4^ |
| No | 373 (74.30%) | 93 (74.40%) | 100 (80.00%) | 86 (68.25%) | 94 (74.60%) |  |
| Yes | 129 (25.70%) | 32 (25.60%) | 25 (20.00%) | 40 (31.75%) | 32 (25.40%) |  |
| **Diabetes** |  |  |  |  |  | 0.916^4^ |
| No | 456 (90.84%) | 113 (90.40%) | 112 (89.60%) | 116 (92.06%) | 115 (91.27%) |  |
| Yes | 46 (9.16%) | 12 (9.60%) | 13 (10.40%) | 10 (7.94%) | 11 (8.73%) |  |
| **Hypertension** |  |  |  |  |  | 0.036^4^ |
| No | 394 (78.49%) | 105 (84.00%) | 94 (75.20%) | 105 (83.33%) | 90 (71.43%) |  |
| Yes | 108 (21.51%) | 20 (16.00%) | 31 (24.80%) | 21 (16.67%) | 36 (28.57%) |  |
| **CHD** |  |  |  |  |  | 0.923^4^ |
| No | 466 (92.83%) | 115 (92.00%) | 117 (93.60%) | 118 (93.65%) | 116 (92.06%) |  |
| Yes | 36 (7.17%) | 10 (8.00%) | 8 (6.40%) | 8 (6.35%) | 10 (7.94%) |  |
| **Surgery** |  |  |  |  |  | 0.004^4^ |
| No | 436 (86.85%) | 97 (77.60%) | 111 (88.80%) | 116 (92.06%) | 112 (88.89%) |  |
| Yes | 66 (13.15%) | 28 (22.40%) | 14 (11.20%) | 10 (7.94%) | 14 (11.11%) |  |
| **Radiotherapy** |  |  |  |  |  | 0.005^4^ |
| No | 413 (82.27%) | 113 (90.40%) | 106 (84.80%) | 101 (80.16%) | 93 (73.81%) |  |
| Yes | 89 (17.73%) | 12 (9.60%) | 19 (15.20%) | 25 (19.84%) | 33 (26.19%) |  |
| **Stage** |  |  |  |  |  | 0.012^4^ |
| Ⅰ stage | 21 (4.18%) | 11 (8.80%) | 3 (2.40%) | 2 (1.59%) | 5 (3.97%) |  |
| Ⅱ stage | 55 (10.96%) | 21 (16.80%) | 13 (10.40%) | 11 (8.73%) | 10 (7.94%) |  |
| Ⅲ stage | 194 (38.65%) | 48 (38.40%) | 46 (36.80%) | 56 (44.44%) | 44 (34.92%) |  |
| Ⅳ stage | 232 (46.22%) | 45 (36.00%) | 63 (50.40%) | 57 (45.24%) | 67 (53.17%) |  |
| **Pleural effusion** |  |  |  |  |  | 0.009^4^ |
| No | 415 (82.67%) | 111 (88.80%) | 108 (86.40%) | 103 (81.75%) | 93 (73.81%) |  |
| Yes | 87 (17.33%) | 14 (11.20%) | 17 (13.60%) | 23 (18.25%) | 33 (26.19%) |  |
| **Chemotherapy regimen** |  |  |  |  |  | 0.031^4^ |
| NPBC | 156 (31.08%) | 36 (28.80%) | 31 (24.80%) | 37 (29.37%) | 52 (41.27%) |  |
| PBC | 346 (68.92%) | 89 (71.20%) | 94 (75.20%) | 89 (70.63%) | 74 (58.73%) |  |

^1^Median (IQR); Mean ± SD; n (%), ^2^Kruskal-Wallis rank sum test, ^3^One-way ANOVA, ^4^Pearson's Chi-squared test.
